# A Web-Based, Time-Use App To Assess Children’s Movement Behaviors: Validation Study of My E-Diary for Activities and Lifestyle (MEDAL)

**DOI:** 10.2196/33312

**Published:** 2022-06-24

**Authors:** Sarah Yi Xuan Tan, Airu Chia, Bee Choo Tai, Padmapriya Natarajan, Claire Marie Jie Lin Goh, Lynette P Shek, Seang Mei Saw, Mary Foong-Fong Chong, Falk Müller-Riemenschneider

**Affiliations:** 1 Saw Swee Hock School of Public Health National University of Singapore and National University Health System Singapore Singapore; 2 Yong Loo Lin School of Medicine National University of Singapore Singapore Singapore; 3 Department of Obstetrics and Gynaecology Yong Loo Lin School of Medicine National University of Singapore Singapore Singapore; 4 Department of Paediatrics Yong Loo Lin School of Medicine National University of Singapore Singapore Singapore; 5 Khoo Teck Puat - National University Children’s Medical Institute National University Health System Singapore Singapore; 6 Singapore Eye Research Institute Singapore National Eye Centre Singapore Singapore; 7 Duke-NUS Medical School Singapore Singapore; 8 Singapore Institute for Clinical Sciences Agency for Science, Technology and Research Singapore Singapore

**Keywords:** children, accelerometer, MEDAL, web-based app, self-report, validity, physical activity, movement behavior, pediatrics, sleep, digital health, behavior

## Abstract

**Background:**

Existing modes of collecting self-reported 24-hour movement information from children, including digital assessments, have not been demonstrated to be of acceptable validity when compared to objective measurements. My E-Diary for Activities and Lifestyle (MEDAL) is an interactive web-based diary developed to collect time-use information from children aged 10 years and older.

**Objective:**

This study evaluated the validity of MEDAL for assessing children’s movement behaviors by comparing self-reported and accelerometer-measured time spent in movement behavior among children in Singapore aged 10-11 years.

**Methods:**

Funding for this study was obtained in October 2017, and data were collected between April and August 2020. Participants recorded their daily activities using MEDAL over 2 specified weekdays and 2 weekend days and wore an Actigraph accelerometer on their nondominant wrist throughout the study to objectively assess movement behaviors. Spearman correlation coefficient and intraclass correlation coefficient (ICC) were used to compare the accelerometer measurements and self-reports for each movement behavior. Bland-Altman plots were generated to investigate trends of bias in the self-reports.

**Results:**

Among the participants aged 10-11 years (29/49, 59% boys), we observed that children reported lower light physical activity (LPA) and higher moderate-to-vigorous physical activity (MVPA), inactivity, and night sleep than that measured by the accelerometer. There was a moderate-to-strong correlation between self-reported and accelerometer-measured MVPA (*r*=0.37; 95% CI 0.20-0.54), inactivity (*r*=0.36; 95% CI 0.18-0.54), and night sleep (*r*=0.58; 95% CI 0.43-0.74); the correlation for LPA was poor (*r*=0.19; 95% CI 0.02-0.36). Agreement was poor for all behaviors (MVPA: ICC=0.24, 95% CI 0.07-0.40; LPA: ICC=0.19, 95% CI 0.01-0.36; inactivity: ICC=0.29, 95% CI 0.11-0.44; night sleep: ICC=0.45, 95% CI 0.29-0.58). There was stronger correlation and agreement on weekdays for inactivity and night sleep; conversely, there was stronger correlation and agreement for MVPA and LPA on weekend days. Finally, based on Bland-Altman plots, we observed that with increasing MVPA, children tended to report higher MVPA than that measured by the accelerometer. There were no clear trends for the other behaviors.

**Conclusions:**

MEDAL may be used to assess the movement behaviors of children. Based on self-reports, the children are able to estimate their time spent in MVPA, inactivity, and night sleep although actual time spent in these behaviors may differ from accelerometer-derived estimates; self-reported LPA warrant cautious interpretation. Observable differences in reporting accuracy exist between weekdays and weekend days.

## Introduction

Identifying trends in children’s time use to address problematic lifestyles has been recognized as a global priority to minimize the burden of noncommunicable diseases in adulthood [1]. As compelling evidence regarding the quality of children and adolescents’ time use and its influence on health emerge, the World Health Organization [2] as well as several countries, including Singapore [3], have developed integrated movement guidelines to reinforce the importance of leading balanced and active lifestyles.

Understanding the lifestyle behaviors of children in relation to the integrated guidelines developed can potentiate the development of targeted behavioral interventions and programs aimed at improving and promoting healthy lifestyles that children can sustain and bring into adulthood. To do so, “adequate, affordable, and convenient” data collection measures to assess 24-hour movement behaviors are required [4].

Self-reported measures (eg, questionnaires, diaries) are commonly used for the collection of information on and assessment of movement behaviors [5,6]. They are inexpensive and easy to administer, however, are subject to the reliance on the respondent’s memory, resulting in recall bias and social desirability bias [5,7]. Children have been found to struggle with reporting durations and intensities of activities [8] and often lack the motivation to complete questionnaires [9], limiting the validity of these measures [4,10,11]. Furthermore, most questionnaires are specialized and focus on single behaviors, often requiring a combination of questionnaires to assess all movement behaviors. Having multiple questionnaires may be tedious for researchers to administer and increases participant burden. There is thus a demand for assessments that can capture the full spectrum of movement behaviors (ie, moderate-to vigorous physical activity [MVPA], light physical activity [LPA], sedentary behavior or inactivity, and sleep).

Objective methods, such as the use of accelerometers, offer a reliable and valid means of objectively capturing 24-hour movement behavior data [12,13]. However, they can be expensive and logistically challenging to administer [14], in that collecting, processing, and analyzing accelerometer data can be complex and require expertise [15]. In addition, accelerometers do not capture contextual information of these movements (eg, type and location of the activities undertaken) [11,14] and are unable to objectively assess screen-viewing, a pertinent behavior with well-established negative associations with the physical and psychosocial outcomes of children [16]. These limitations impede the understanding of children’s behaviors and consequentially limit the development of targeted behavioral interventions.

The limitations of existing methods that assess movement behaviors warrant a need for a valid, low-burden, and cost-efficient data collection method to collect 24-hour movement information from children. Advancements in technology may circumvent some of the challenges of existing data collection methods, yet few digital assessments exist. Multimedia activity recall for children and adolescents (MARCA) [9], Synchronised Nutrition and Activity Program (SNAP) [17], and MyDailyMoves [4] are digital assessments developed to provide ease of collecting self-reported 24-hour movement behavior information from children. However, not all self-reported behaviors reported on these assessments have been validated against objective measurements; therefore, the utility of these assessments for collecting 24-hour movement behavior information in comparison to objective measures remains unclear. These applications were also developed for Western populations (ie, Australia, the United Kingdom, and the Netherlands, respectively), and the behavioral patterns of children in Western versus Asian populations may differ (eg, the prevalence of children attending tuition or shadow education) [18], limiting the relevance of existing applications to the Asian setting.

To bridge this gap, My E-Diary for Activities and Lifestyle (MEDAL), an interactive web-based diary, was developed to collect time-use information from children of at least 10 years of age in Singapore [18]. Usability testing suggests that MEDAL is a feasible application for capturing the movement behaviors of children aged 10 to 12 years [18]. We aimed to validate the use of MEDAL for assessing children’s movement behaviors by comparing self-reported and accelerometer-measured time spent in MVPA, LPA, inactivity, and sleep among children aged 10 to 11 years in Singapore. We hypothesized that children at this age would be able to self- report their movement behaviors on MEDAL accurately although some differences between self-reported and objective measures would be expected.

## Methods

### Study Participants

Boys and girls aged 10 to 11 years (Primary 5 level) from 2 government schools in Singapore (referred to as schools A and B in the present study) were recruited between April and August 2020.

Of the 7 and 6 Primary 5 classes from schools A and B, respectively, 2 classes from each school (35 to 41 students per class) underwent convenience sampling to participate in this study based on logistical feasibility (ie, ease of administration of accelerometers). Students of the remaining classes were involved in the validation of other self-reported variables (ie, diet and outdoor time).

Funding for this study was obtained in October 2017, and data were collected between April and August 2020.

### Ethics Approval and Consent To Participate

The Singapore Ministry of Education approved of the collection of data from schools A and B, and the National University of Singapore Institutional Review Board (reference code #S-18-088) approved of the study. Written informed consent was obtained from parents or guardians, and all participants provided verbal assent.

### Data Collection and Processing

#### Assessment of Movement Behaviors

A demonstration session was conducted, where trained researchers demonstrated the use of and navigations in MEDAL. Participants were instructed to record the diet and activities that they engaged in from midnight to midnight of the recording day at home over 2 specified weekdays and 2 weekend days. A special arrangement was made for 1 participant in this study without access to the internet at home to complete his or her recording on MEDAL using the school computer.

The details and features of MEDAL have been reported elsewhere [18]. In brief, participants were instructed to enter the time they slept the previous day, the time they woke up, and all the activities in which they participated in chronological order until the time they went to bed. Participants could select from 6 broad activities: “Shower/Wash Up,” “Travelling,” “Eat & Drink,” “Nap/Sleep,” “Sitting Activities,” or “Active Activities” and were prompted to specify the mode of transport, types of sitting and active activities engaged in, and what they ate or drank. Participants were also allowed to select concurrent activities that occurred while engaged in “Travelling,” “Eat & Drink,” or “Sitting Activities.” When “Active Activities” was selected, the participants were prompted to report their perceived intensity of the activity based on the “Talk Test” [19]. Selecting “Just a little tiring—You can sing and talk during the activity” indicated that the activity was of light intensity, “Quite tiring—You can talk but cannot sing during the activity” indicated that the activity was of moderate intensity, and “Very tiring—You cannot say more than a few words without pausing” indicated that the activity was of vigorous intensity. All other activity entries (ie, excluding “Active Activities”) were coded as “night sleep,” “inactivity,” “light physical activity,” or “moderate-to-vigorous physical activity” based on previously established metabolic intensities [20] (Multimedia Appendix 1). All information collected was secure, in that only the investigators had access to the password-protected data.

Each participant involved in the movement behavior validation study was attached with a triaxial accelerometer (Actigraph wGT3X-BT) using a nonremovable strap on their nondominant wrist during the demonstration session. The accelerometers were initialized to start recording raw acceleration data at a rate of 80 Hz from midnight of the day after the demonstration session. The participants were instructed to wear the accelerometers at all times, even when sleeping, for 6 to 7 days, which overlapped with the days that they were instructed to record on MEDAL. They were only advised to remove the accelerometers by cutting the nonremovable strap when they engaged in any activity that might have involved physical contact or when the wearing of devices was not allowed (eg, sports competitions). They were required to record the date and time the accelerometers were removed and were instructed to reattach the accelerometer after the activity using a spare strap provided. This allowed the objective measurement of their movement behaviors throughout the study period to validate their self-reported activities on MEDAL.

Data processing was conducted using an established protocol [21]. Raw data were downloaded using the ActiLife software (version 6.13.4) in GT3X format and processed in R version 4.0.2 (The R Project for Statistical Computing) with the GGIR package (version 2.0-0). After the raw accelerometer signals were auto-calibrated and converted into gravity-corrected vector magnitude units (Euclidean Norm Minus One [ENMO]), a wear time inclusion criterion of a minimum of 16 hours per day for at least 3 days was applied [21]. Nonwear time was detected using information on the SD and value range of each accelerometer axis at 60-minute windows in 15-minute increments. Accelerometer wear compliance was assessed for valid accelerometer wear days that corresponded with the day of MEDAL recording. Sleep duration was assessed using the method developed by van Hees and colleagues [22]. The term “sedentary behavior,” by definition refers to activities “≤1.5 METs [metabolic equivalents] while in a sitting, reclining or lying posture” [23]. As wrist-worn accelerometers are unable to determine the posture of participants, the term “inactivity” was used as proxy for sedentary behaviors in this study. With that, activities during waking hours were classified as inactivity (ENMO <35.0 mg), LPA (ENMO 35.0-200 mg), or MVPA (ENMO >200.0 mg) based on acceleration thresholds developed for children aged 7 to 11 years by Hildebrand and colleagues [24,25], which have been applied in previous studies [21,26,27].

#### Demographic Data Collection

During the first sign-in on MEDAL, participants were prompted to report their age and sex information. Primary schools in Singapore routinely measure the students’ height and weight to monitor growth. The most recent height and weight measurements taken by the school teachers were shared by the participating schools. BMI was calculated using the formula: weight (kg)/(height [m] × height [m]). The subsequent value was then classified as underweight (<5th percentile), healthy weight (5th to <90th percentile), and overweight (≥90th percentile) based on the age- and sex-specific BMI reference data for Singaporean children [28].

### Statistical Analyses

MEDAL entries with implausible values reported were excluded from the analysis. Additionally, each participant’s graphical acceleration data were visually inspected, and days where sleep period appeared inaccurate (eg, implausibly short sleep duration, early wake-up, or long interruptions during sleep period) were excluded, as the estimation of time spent in all movement behaviors would be affected. Descriptive statistics are presented as frequencies, percentages, or medians with IQR. The difference in distribution of characteristics between included and excluded participants was assessed by Fisher exact test. The correlation between accelerometer-measured and self-reported daily time spent in movement behavior (ie, MVPA, LPA, inactivity, and night sleep) were calculated using Spearman correlation coefficient test. Spearman correlation coefficients were interpreted as a poor (≤0.29), moderate (0.30-0.39), strong (0.40-0.69), and very strong correlation (≥0.70) [29]. Intraclass correlation coefficients (ICCs) were calculated to quantify the agreement between accelerometer-measured and self-reported daily time spent in each movement behavior (ie, MVPA, LPA, inactivity, and night sleep) using 2-way mixed-effects models. Based on previously established cutoffs [30], the strength of agreement was interpreted as poor (<0.50), moderate (0.50-0.74), good (0.75-0.90), or excellent (>0.90). Bland-Altman plots, which accounted for repeated measurements [31], were generated with 95% limits of agreement (LoA) to visualize agreement and investigate trends of bias in the self-reports when compared to accelerometer measurements. All statistical tests were performed using Stata Special Edition version 14.2 (StataCorp). All evaluations were made assuming a 2-sided test at a 5% level of significance.

## Results

### Study Participants

**Figure 1:**
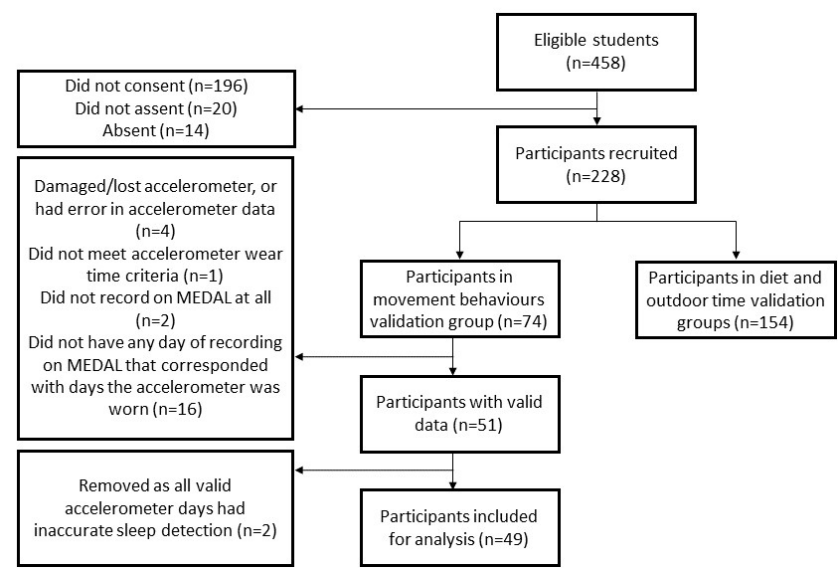
MEDAL movement behaviors validation participant flow diagram. MEDAL: My E-Diary for Activities and Lifestyle (validation study).

There were 74 participants (48 participants and 26 participants from schools A and B, respectively) who took part in the movement behavior validation study, of whom 49 were included in the analysis (66%). Figure 1 summarizes the participant flow diagram of this study.

Participants were mostly males (29/49, 59%), of healthy weight (33/49, 69%), and had access to the internet (43/49, 98%). Compliance with accelerometer wear among included participants was excellent: 47 out of 49 participants (96%) had at least 90% valid accelerometer wear time per day (data not shown). Table 1 summarizes the demographic and compliance information of our sample.

**Table 1:** Demographic characteristics and accelerometer wear time of Primary 5 students participating in the My E-Diary for Activities and Lifestyle (MEDAL) movement behaviors validation study (N=49).

			Participants (N=49)^a^
**Age, n (%)**			
	10 years	20 (41)	
	11 years	28 (57)	
	12 years	1 (2)	
**Sex, n (%)**			
	Females	20 (41)	
	Males	29 (59)	
**BMI for age, n (%)**			
	Underweight (<5th percentile)	10 (21)	
	Healthy (5th to <90th percentile)	33 (69)	
	Overweight (≥90th percentile)	5 (10)	
**Internet access, n (%)**			
	Yes	43 (98)	
	No	1 (2)	
Valid accelerometer wear time (hours/day), median (IQR)			24.0 (23.7-24.0)

^a^One participant had missing BMI-for-age information, and five participants had missing information on internet access.

### Accelerometer-Measured and Self-Reported Movement Behavior Durations

Table 2 presents the objectively measured and self-reported time spent in each movement behavior on weekdays and on weekend days.

In comparison to accelerometer measurements, the participants reported lower LPA (312.1 min/day vs 52.5 min/day) and higher MVPA (53.5 min/day vs 60.0 min/day), inactivity (567.2 min/day vs 742.5 min/day), and night sleep durations (488.8 min/day vs 545.0 min/day) in MEDAL. Accelerometer-measured and self-reported MVPA, LPA, and inactivity on weekdays were higher than on weekend days, whereas accelerometer-measured and self-reported night sleep on weekend days was higher than on weekdays.

**Table 2:** Median accelerometer-measured and self-reported time spent in movement behaviors on average of all days, weekdays, and weekend days (minutes/day).

Type of movement behavior	Average of all days (m/d^a^), median (IQR) (n=49)			Weekdays (m/d), median (IQR) (n=41)			Weekend days (m/d), median (IQR) (n=45)	
	Accelerometer (IQR)	MEDAL (IQR)	Accelerometer (IQR)		MEDAL^b^ (IQR)	Accelerometer (IQR)		MEDAL (IQR)
MVPA^c^	53.5 (37.3-77.6)	60.0 (0.0-120.0)	62.3 (43.5-86.5)		70.0 (32.5-150.0)	47.3 (32.8-73.0)		42.5 (0.0-120.0)
LPA^d^	312.1 (257.7-356.6)	52.5 (30.0-95.0)	345.2 (297.5-390.2)		55.0 (32.5-77.5)	297.8 (243.3-328.7)		45.0 (27.5-130.0)
Inactivity	567.2 (510.5-640.3)	742.5 (650.0-807.5)	612.4 (539.8-657.0)		767.5 (720.0-870.0)	547.0 (495.2-604.6)		715.0 (587.5-780.0)
Night sleep	488.8 (432.5-556.5)	545.0 (485.0-612.5)	431.4 (389.2-468.6)		492.5 (435.0-530.0)	546.1 (488.5-582.8)		595.0 (540.0-657.5)

^a^m/d: minutes per day.

^b^MEDAL: My E-Diary for Activities and Lifestyle.

^c^MVPA: moderate-to-vigorous physical activity.

^d^LPA: light physical activity.

### Correlation Between Accelerometer-Measured and Self-Reported Movement Behavior Durations

Spearman correlation tests revealed a strong correlation between accelerometer-measured and self-reported night sleep (*r*=0.58; 95% CI 0.43-0.74). There was a moderate correlation for MVPA (*r*=0.37; 95% CI 0.20-0.54) and inactivity (*r*=0.36; 95% CI 0.18-0.54), and a poor correlation for LPA (*r*=0.19; 95% CI 0.02-0.36). Correlation analyses stratified by weekdays and weekend days revealed that only weekend days’ MVPA (*r*=0.44; 95% CI 0.23-0.65) and LPA (*r*=0.33; 95% CI 0.10-0.56) and weekday inactivity (*r*=0.36; 95% CI 0.05-0.66) and night sleep (*r*=0.64; 95% CI 0.45-0.84) demonstrated a moderate-to-strong correlation between accelerometer-measured and self-reported values. These results are presented in Table 3.

**Table 3:** Spearman correlation and ICC between accelerometer-measured and self-reported time spent in each movement behavior.

Type of movement behavior	Average of all days (n=49)		Weekdays (n=41)			Weekend days (n=45)	
	*r* (95% CI)^a^	ICC^b^ (95% CI)^c^	*r* (95% CI)	ICC (95% CI)	*r* (95% CI)		ICC (95% CI)
MVPA^d^	0.37 (0.20 to 0.54)	0.24 (0.07 to 0.40)	0.15 (–0.16 to 0.47)	0.05 (–0.23 to 0.33)	0.44 (0.23 to 0.65)		0.35 (0.13 to 0.53)
LPA^e^	0.19 (0.02 to 0.36)	0.19 (0.01 to 0.36)	0.01 (–0.27 to 0.30)	0.10 (–0.18 to 0.37)	0.33 (0.10 to 0.56)		0.32 (0.09 to 0.51)
Inactivity	0.36 (0.18 to 0.54)	0.29 (0.11 to 0.44)	0.36 (0.05 to 0.66)	0.32 (0.05 to 0.55)	0.32 (0.09 to 0.55)		0.18 (–0.05 to 0.40)
Night sleep	0.58 (0.43 to 0.74)	0.45 (0.29 to 0.58)	0.64 (0.45 to 0.84)	0.44 (0.18 to 0.64)	0.25 (–0.01 to 0.50)		0.11 (–0.13 to 0.33)

^a^Spearman correlation coefficients (*r*) were interpreted as poor (≤0.29), moderate (0.30-0.39), strong (0.40-0.69), or very strong (≥0.70) correlations.

^b^ICC: intraclass correlation coefficient.

^c^ICCs were interpreted as poor (<0.50), moderate (0.50-0.74), good (0.75-0.90), or excellent (>0.90) agreement.

^d^MVPA: moderate-to-vigorous physical activity.

^e^LPA: light physical activity.

### Agreement Between Accelerometer-Measured and Self-Reported Movement Behavior Durations

Agreement was poor between the measures for MVPA (ICC=0.24; 95% CI 0.07-0.40), LPA (ICC=0.19; 95% CI 0.01-0.36), inactivity (ICC=0.29; 95% CI 0.11-0.44), and night sleep (ICC=0.45; 95% CI 0.29-0.58). ICC analyses stratified by weekdays and weekend days demonstrated that there was poor agreement for both weekdays and weekend days in MVPA, LPA, inactivity, and night sleep. These results are presented in Table 3.

**Figure 2:**
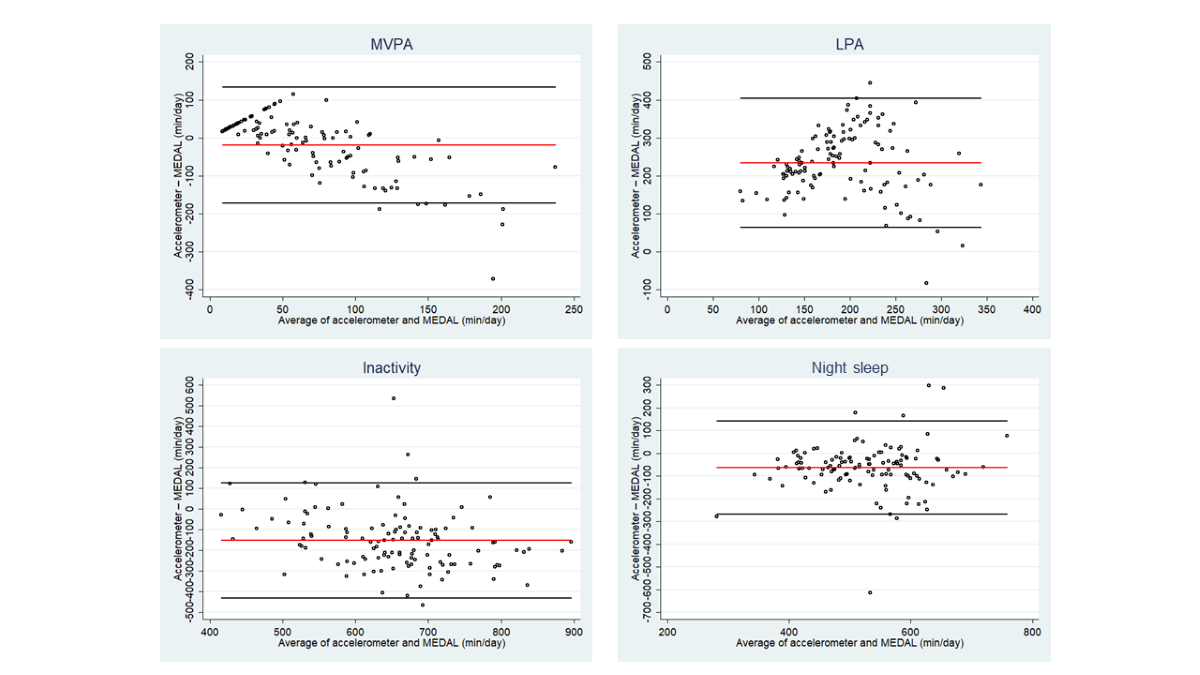
Bland-Altman plots between accelerometer-measured and self-reported (ie, MEDAL) MVPA, LPA, inactivity, and night sleep based on the average of all days. The black lines depict the 95% limits of agreement, and the red line depicts the mean difference. LPA: light physical activity; MEDAL: My E-Diary for Activities and Lifestyle (validation study); MVPA: moderate-to-vigorous physical activity.

The Bland-Altman plots (Figure 2) suggested that those with low MVPA (based on average of self-reported and accelerometer-measured MVPA) reported lower MVPA levels than those measured by the accelerometer. As MVPA levels increased, reporting higher MVPA levels than those measured by the accelerometer was more common.

There were no clear trends for the other movement behaviors (ie, LPA, inactivity, and night sleep), as the plots appeared to be randomly distributed. Based on these plots, self-reported LPA was on average 234.1 minutes lower than that measured by the accelerometer (95% LoA 63.4-404.8 minutes). On the other hand, accelerometer-measured inactivity was on average 151.5 minutes lower than self-reported activity (95% LoA –430.5 to 127.6 minutes), and accelerometer-measured night sleep was on average 62.6 minutes lower than the self-reported night sleep (95% LoA –266.6 to 141.4 minutes). The plots stratified by weekday and weekend days did not differ meaningfully (figures not shown).

## Discussion

In this study, we evaluated the validity of MEDAL by comparing self-reported time spent in each movement behavior reported (ie, MVPA, LPA, inactivity, and sleep) on MEDAL to accelerometer measurements among children in Singapore aged 10 to 11 years. We found that the children reported lower LPA, higher MVPA, higher inactivity, and higher night sleep durations than those measured by the accelerometer. Moderate-to-strong correlations were observed between accelerometer-measured and self-reported MVPA, inactivity, and night sleep, while LPA demonstrated a poor correlation. There was generally poor agreement between accelerometer-measured and self-reported values for all behaviors. Differences between weekdays and weekend days in correlation and agreement between self-reports and accelerometer measurements were also observed.

We demonstrated that some movement behaviors were better reported on MEDAL compared to others. In particular, LPA was most poorly reported, as it had the lowest correlation with accelerometer measurements, and a similar strength of correlation was reported previously (*r*=0.09) [32]. The literature suggests that it is difficult to define LPA and, consequentially, difficult to capture LPA using subjective assessments such as self-reports, especially among children [33]. This is attributed to the sporadic and spontaneous nature of activity among children of this age [14]; periods of light activity may be intermittent in nature, which is attributable to children’s short attention span and quick disinterest in continuous activity. These short bursts of light activity contribute to differences in objectively measured and self-reported physical activity and inactivity durations, as short periods of movement or inactivity might not be salient during their recall and thus, unintentionally misreported. The strength of associations between self-reported and accelerometer-measured MVPA and inactivity was similar or better than that reported in other studies, respectively. Recent systematic reviews [10,11] found that self-reported MVPA durations among children aged around 10 to 11 years had moderate correlation with objective measurements (*r*=0.21-0.48) [32,34-37], whereas correlations were poor for self-reported inactivity (*r*=0.06-0.14) [35,38]. Remembering frequencies, durations, and intensities of all activities undertaken in a day is difficult for children [8], contributing to the recall bias that underlies observable differences in time spent in each movement behavior between objectively measured and self-reported measures.

Lastly, for sleep, we demonstrated that night sleep was better reported on MEDAL compared to the other behaviors. Based on a systematic review of sleep questionnaires developed for children and adolescents [39], the criteria for assessing sleep duration in previous studies differ from the current one, and thus the ability to make comparisons to other studies was limited. Notwithstanding, it has been established that sleep latency, defined as the time a person takes to fall asleep after going to bed, may influence reporting of sleep times [40]. Periods between bed time and falling asleep might be reported as sleeping but detected from accelerometer data to be otherwise, resulting in differences between objectively measured and self-reported sleep onset time and, consequentially, total sleep time.

Previous studies have typically only investigated the correlation between objective measurements and self-reports, which is a limitation when assessing validity, as correlation does not provide information on the strength of agreement between the methods [41]. In our study, we examined agreement and found that while MVPA, inactivity, and night sleep demonstrated moderate-to-strong correlations between accelerometer measurements and self-reports on MEDAL, agreement for all behaviors was poor. To our knowledge, only MVPA has been examined previously in terms of agreement between objective measurements and self-reports [37,42]. The findings of our study were in line with these studies (ICC=0.06-0.25) in that agreement was poor. Although children are able to report some behaviors using MEDAL, actual time spent in these behaviors may differ from estimates by wrist-worn accelerometers.

Finally, we investigated whether the validity of MEDAL differed on weekdays and weekend days. This adds to the existing literature, as few studies have investigated differences in reporting accuracy on weekdays versus weekend days. Our study suggests that the children were able to report weekend days’ physical activity better than weekdays’; conversely, they were able to report weekdays’ inactivity and night sleep better than weekend days’. The greater correlation and agreement for inactivity and night sleep demonstrated on weekdays might be attributed to weekdays being more structured, characterized by consistent wake and bed times [43] as well as regular school-day routines in which the majority of activities are inactive (eg, lessons in school) [44], making them easier to recall. Explanations for differences in reporting accuracy for physical activity (MVPA and LPA) on weekdays compared to weekend days are less clear. Participants might have reported their physical education lessons or after-school sports trainings on weekdays as entirely physical activity (regardless of MVPA or LPA). In reality, these periods of “activity” might include organizing the lesson or game, watching demonstrations, or taking turns to rest and play [33]. The accelerometer only measured the amount of time when the individual was actually moving, resulting in the discrepancy between periods of physical activity reported on MEDAL and those measured by the accelerometers. Conversely, the participants might have participated in a specific sport and might have been active throughout the duration of the physical activity they reported, resulting in the greater correlation and agreement between accelerometer-measured and self-reported physical activity durations on weekend days. This hypothesis warrants further investigation.

Despite known limitations of self-reports relating to recall bias, self-reporting offers advantages that objective assessments of movement behaviors, like use of accelerometers, do not [6]. Objective assessments are independent of recall bias, while self-reported measures, like MEDAL, are easier to administer, process, and analyze, particularly when extended to population-based studies or large cohorts [45]. They also offer the potential of collecting information on screen-viewing and contextual information, such as location and type of activity, without the need for complementary questionnaires or devices (eg, a location tracker) [45], streamlining the assessment and identification of problematic health behaviors and possible contexts to target health behavior interventions. Hence, the impetus for selecting an appropriate method to assess movement behaviors, whether self-reports or objective assessments or a combination of both, is dependent on the research question [45].

Our findings suggest that based on self-reports on MEDAL, the children are able to estimate their time spent in MVPA, inactivity, and night sleep although actual time spent in these behaviors may differ from estimates by wrist-worn accelerometers. On the other hand, self-reported LPA on MEDAL warrants cautious interpretation. Finally, MVPA and LPA might be better reported by children on weekend days, and inactivity and night sleep might be better reported on weekdays.

There are several strengths of the present study. High compliance with accelerometer wear allowed activity patterns of a full or close to a full 24-hour day to be objectively measured for comparison with self-reports on MEDAL. The reporting of all 4 movement behaviors was investigated, allowing us to review the validity of MEDAL as a tool for capturing time use across the full movement spectrum of 24 hours. Stratifying the analyses by weekdays and weekend days provided additional interesting findings to contribute to the current literature.

However, there are limitations to be acknowledged. First, we note that some participants might have removed their accelerometers during certain sports and activities (eg, contact sports, martial arts); therefore, accelerometer-measured MVPA and LPA might have been underestimated. As our results demonstrate that participants reported much lower LPA than did the accelerometer, the underreporting may be more pronounced than reflected. Sleep duration detection algorithms for raw acceleration data have only been developed to detect the onset of sleep [22], which may differ from bed time and would be intuitively self-reported. The difference in time between sleep onset (ie, when the participant truly falls asleep) and bed time (ie, the time the participant goes to bed to go to sleep) would consequentially influence estimations of sleep durations. As accelerometers and MEDAL assessed different constructs of sleep (ie, sleep onset to wake time versus bed time to wake time), accelerometers might be less adequate as a means of comparison against self-reported sleep durations. We also removed 1 MEDAL entry of a participant whom we deemed to have reported implausible values. Sensitivity analyses, however, revealed that including the entry did not affect results substantially. We also acknowledge that the sample size of this study was modest, largely due to the exclusion of participants that did not have any day of recording on MEDAL that corresponded with the days the accelerometers were worn. Notwithstanding, MEDAL was reported previously to be feasible and acceptable among children of this age group [18]. Some differences in characteristics exist between participants included in our study and those recruited but not included (ie, those excluded from movement behavior validation analyses and those involved in the other validation studies; Multimedia Appendix 2). These may potentially limit the generalizability of the findings of this study to the population.

This is among the first studies to assess the validity of self-reported behaviors across the full movement spectrum and to compare reporting accuracy on weekdays versus weekend days. This study suggests that MEDAL may be useful in assessing movement behaviors of children aged 10 to 11 years although estimates may differ from wrist-worn accelerometer measurements. Self-reported estimates for inactivity and sleep on weekdays might be more accurate than those on weekend days, whereas self-reported estimates for MVPA and LPA on weekend days might be more accurate than those on weekdays. Findings of this study will facilitate the interpretation of future data collected using MEDAL.

## Appendix

Multimedia Appendix 1 Classification of activities reported on My E-Diary for Activities and Lifestyle (MEDAL) as inactivity, light physical activity, moderate-to-vigorous physical activity, and sleep.

Multimedia Appendix 2 Demographic characteristics of Primary 5 students included and those recruited but not included in the My E-Diary for Activities and Lifestyle (MEDAL) movement behaviors validation study.

## Abbreviations

ENMO: Euclidean Norm Minus One
ICC: intraclass correlation coefficient
LoA: limits of agreement
LPA: light physical activity
MARCA: multimedia activity recall for children and adolescents
MEDAL: My E-Diary for Activities and Lifestyle
MET: metabolic equivalents
MVPA: moderate-to-vigorous physical activity
NUHS: National University Health System.
NUS: National University of Singapore.
SNAP: Synchronised Nutrition and Activity Program

## Data Availability

The data sets used and analyzed during this study are available from the corresponding author on reasonable request.

## References

[1] Prioritizing areas for action in the field of population-based prevention of childhood obesity 2012. World Health Organization.

[2] Chaput J, Willumsen J, Bull F, Chou R, Ekelund U, Firth J, Jago R, Ortega FB, Katzmarzyk PT (2020). 2020 WHO guidelines on physical activity and sedentary behaviour for children and adolescents aged 5–17 years: summary of the evidence. Int J Behav Nutr Phys Act.

[3] College of Paediatrics & Children Health Singapore.

[4] Hidding LM, Chinapaw MJM, Belmon LS, Altenburg TM (2020). Co-creating a 24-hour movement behavior tool together with 9-12-year-old children using mixed-methods: MyDailyMoves. Int J Behav Nutr Phys Act.

[5] Sallis J (1991). Self-report measures of children's physical activity. J Sch Health.

[6] Ferrari GLDM, Kovalskys I, Fisberg M, Gómez Georgina, Rigotti A, Sanabria LYC, García Martha Cecilia Yépez, Torres RGP, Herrera-Cuenca M, Zimberg IZ, Guajardo V, Pratt M, Pires CAM, Colley RC, Solé Dirceu, ELANS Study Group (2020). Comparison of self-report versus accelerometer - measured physical activity and sedentary behaviors and their association with body composition in Latin American countries. PLoS One.

[7] Ainsworth B, Cahalin L, Buman M, Ross R (2015). The current state of physical activity assessment tools. Prog Cardiovasc Dis.

[8] Sirard JR, Pate RR (2001). Physical activity assessment in children and adolescents. Sports Med.

[9] Ridley K, Olds TS, Hill A (2006). The multimedia activity recall for children and adolescents (MARCA): development and evaluation. Int J Behav Nutr Phys Act.

[10] Hidding LM, Chinapaw MJM, van Poppel MNM, Mokkink LB, Altenburg TM (2018). An updated systematic review of childhood physical activity questionnaires. Sports Med.

[11] Hidding LM, Altenburg TM, Mokkink LB, Terwee CB, Chinapaw MJM (2017). Systematic Review of Childhood Sedentary Behavior Questionnaires: What do We Know and What is Next?. Sports Med.

[12] Trost S, McIver Kerry L, Pate R (2005). Conducting accelerometer-based activity assessments in field-based research. Med Sci Sports Exerc.

[13] Lubans D, Hesketh K, Cliff D, Barnett L, Salmon J, Dollman J, Morgan P J, Hills A P, Hardy L L (2011). A systematic review of the validity and reliability of sedentary behaviour measures used with children and adolescents. Obes Rev.

[14] Welk GJ, Corbin CB, Dale D (2000). Measurement issues in the assessment of physical activity in children. Res Q Exerc Sport.

[15] Toftager M, Kristensen P, Oliver M, Duncan S, Christiansen L, Boyle E, Brønd Jan Christian, Troelsen J (2013). Accelerometer data reduction in adolescents: effects on sample retention and bias. Int J Behav Nutr Phys Act.

[16] Saunders TJ, Vallance JK (2017). Screen Time and Health Indicators Among Children and Youth: Current Evidence, Limitations and Future Directions. Appl Health Econ Health Policy.

[17] Moore HJ, Ells LJ, McLure SA, Crooks S, Cumbor D, Summerbell CD, Batterham AM (2008). The development and evaluation of a novel computer program to assess previous-day dietary and physical activity behaviours in school children: the Synchronised Nutrition and Activity Program (SNAP). Br J Nutr.

[18] Chia A, Chew MNJS, Tan SYX, Chan MJ, T Colega M, Toh JY, Natarajan P, Lança Carla, Shek LP, Saw S, Müller-Riemenschneider Falk, Chong MF (2021). A Web-Based Time-Use Application to Assess Diet and Movement Behavior in Asian Schoolchildren: Development and Usability Study of My E-Diary for Activities and Lifestyle (MEDAL). J Med Internet Res.

[19] Measuring physical activity intensity 2020. Centers for Disease Control and Prevention.

[20] Butte N, Watson K, Ridley K, Zakeri I, McMurray R, Pfeiffer K, Crouter Scott E, Herrmann Stephen D, Bassett David R, Long Alexander, Berhane Zekarias, Trost Stewart G, Ainsworth Barbara E, Berrigan David, Fulton Janet E (2018). A Youth Compendium of Physical Activities: Activity Codes and Metabolic Intensities. Med Sci Sports Exerc.

[21] Chen B, Bernard JY, Padmapriya N, Yao J, Goh C, Tan KH, Yap F, Chong Y, Shek L, Godfrey KM, Chan S, Eriksson JG, Müller-Riemenschneider Falk (2019). Socio-demographic and maternal predictors of adherence to 24-hour movement guidelines in Singaporean children. Int J Behav Nutr Phys Act.

[22] van Hees VT, Sabia S, Anderson KN, Denton SJ, Oliver J, Catt M, Abell JG, Kivimäki Mika, Trenell MI, Singh-Manoux A (2015). A Novel, Open Access Method to Assess Sleep Duration Using a Wrist-Worn Accelerometer. PLoS One.

[23] Tremblay MS, Aubert S, Barnes JD, Saunders TJ, Carson V, Latimer-Cheung AE, Chastin SF, Altenburg TM, Chinapaw MJ, SBRN Terminology Consensus Project Participants (2017). Sedentary Behavior Research Network (SBRN) - Terminology Consensus Project process and outcome. Int J Behav Nutr Phys Act.

[24] Hildebrand M, van Hees Vincent T, Hansen Bjorge Hermann, Ekelund Ulf (2014). Age group comparability of raw accelerometer output from wrist- and hip-worn monitors. Med Sci Sports Exerc.

[25] Hildebrand M, Hansen BH, van Hees VT, Ekelund U (2017). Evaluation of raw acceleration sedentary thresholds in children and adults. Scand J Med Sci Sports.

[26] Fairclough SJ, Taylor S, Rowlands AV, Boddy LM, Noonan RJ (2019). Average acceleration and intensity gradient of primary school children and associations with indicators of health and well-being. J Sports Sci.

[27] Migueles JH, Cadenas-Sanchez Cristina, Tudor-Locke Catrine, Löf Marie, Esteban-Cornejo Irene, Molina-Garcia Pablo, Mora-Gonzalez Jose, Rodriguez-Ayllon Maria, Garcia-Marmol Eduardo, Ekelund U, Ortega FB (2019). Comparability of published cut-points for the assessment of physical activity: Implications for data harmonization. Scand J Med Sci Sports.

[28] Healthy weight healthy children Singapore 2015. Health Promotion Board.

[29] Cohen J (1988). Statistical power Analysis for the Behavioral Sciences. 2nd ed.

[30] Koo TK, Li MY (2016). A Guideline of Selecting and Reporting Intraclass Correlation Coefficients for Reliability Research. J Chiropr Med.

[31] Bland JM, Altman DG (2007). Agreement between methods of measurement with multiple observations per individual. J Biopharm Stat.

[32] Sprengeler O, Wirsik N, Hebestreit A, Herrmann D, Ahrens W (2017). Domain-specific self-reported and objectively measured physical activity in children. Int J Environ Res Public Health.

[33] Mindell JS, Coombs N, Stamatakis E (2014). Measuring physical activity in children and adolescents for dietary surveys: practicalities, problems and pitfalls. Proc Nutr Soc.

[34] Zelener Jacqueline, Schneider Margaret (2016). Adolescents and Self-Reported Physical Activity: An Evaluation of the Modified Godin Leisure-Time Exercise Questionnaire. Int J Exerc Sci.

[35] Huang Y, Wong S, Salmon J (2009). Reliability and validity of the modified Chinese version of the Children's Leisure Activities Study Survey (CLASS) questionnaire in assessing physical activity among Hong Kong children. Pediatr Exerc Sci.

[36] Määttä Suvi, Nuutinen T, Ray C, Eriksson JG, Weiderpass E, Roos E (2016). Validity of self-reported out-of-school physical activity among Finnish 11-year-old children. Arch Public Health.

[37] Gwynn J, Hardy L, Wiggers J, Smith W, D'Este C, Turner N, Cochrane Janine, Barker Daniel J, Attia John R (2010). The validation of a self-report measure and physical activity of Australian Aboriginal and Torres Strait Islander and non-Indigenous rural children. Aust N Z J Public Health.

[38] Affuso O, Stevens J, Catellier D, McMurray RG, Ward DS, Lytle L, Sothern MS, Young DR (2011). Validity of self-reported leisure-time sedentary behavior in adolescents. J Negat Results Biomed.

[39] Nascimento-Ferreira MV, Collese TS, de Moraes ACF, Rendo-Urteaga T, Moreno LA, Carvalho HB (2016). Validity and reliability of sleep time questionnaires in children and adolescents: A systematic review and meta-analysis. Sleep Med Rev.

[40] Tremaine R, Dorrian J, Blunden S (2010). Subjective and objective sleep in children and adolescents: Measurement, age, and gender differences. Sleep and Biological Rhythms.

[41] Prince SA, Adamo KB, Hamel M, Hardt J, Connor Gorber S, Tremblay M (2008). A comparison of direct versus self-report measures for assessing physical activity in adults: a systematic review. Int J Behav Nutr Phys Act.

[42] Ayala-Guzmán CI, Ramos-Ibáñez N, Ortiz-Hernández L (2017). Accelerometry does not match with self-reported physical activity and sedentary behaviors in Mexican children. Boletín Médico Del Hospital Infantil de México (English Edition).

[43] Spruyt K, Molfese D, Gozal D (2011). Sleep duration, sleep regularity, body weight, and metabolic homeostasis in school-aged children. Pediatrics.

[44] Brazendale K, Beets MW, Armstrong B, Weaver RG, Hunt ET, Pate RR, Brusseau TA, Bohnert AM, Olds T, Tassitano RM, Tenorio MCM, Garcia J, Andersen LB, Davey R, Hallal PC, Jago R, Kolle E, Kriemler S, Kristensen PL, Kwon S, Puder JJ, Salmon J, Sardinha LB, van Sluijs EMF, International Children’s Accelerometry Database (ICAD) Collaborators (2021). Children's moderate-to-vigorous physical activity on weekdays versus weekend days: a multi-country analysis. Int J Behav Nutr Phys Act.

[45] Dollman J, Okely AD, Hardy L, Timperio A, Salmon J, Hills AP (2009). A hitchhiker's guide to assessing young people's physical activity: Deciding what method to use. J Sci Med Sport.

