# Supplementary material for: A Web-Based, Time-Use App To Assess Children’s Movement Behaviors: Validation Study of My E-Diary for Activities and Lifestyle (MEDAL)
Source: JMIR Pediatr Parent. 2022 Jun 24;5(2):e33312. doi: 10.2196/33312 (PMC9270708; doi:10.2196/33312)
Supplement: Multimedia Appendix 1 [file pediatrics_v5i2e33312_app1.docx]

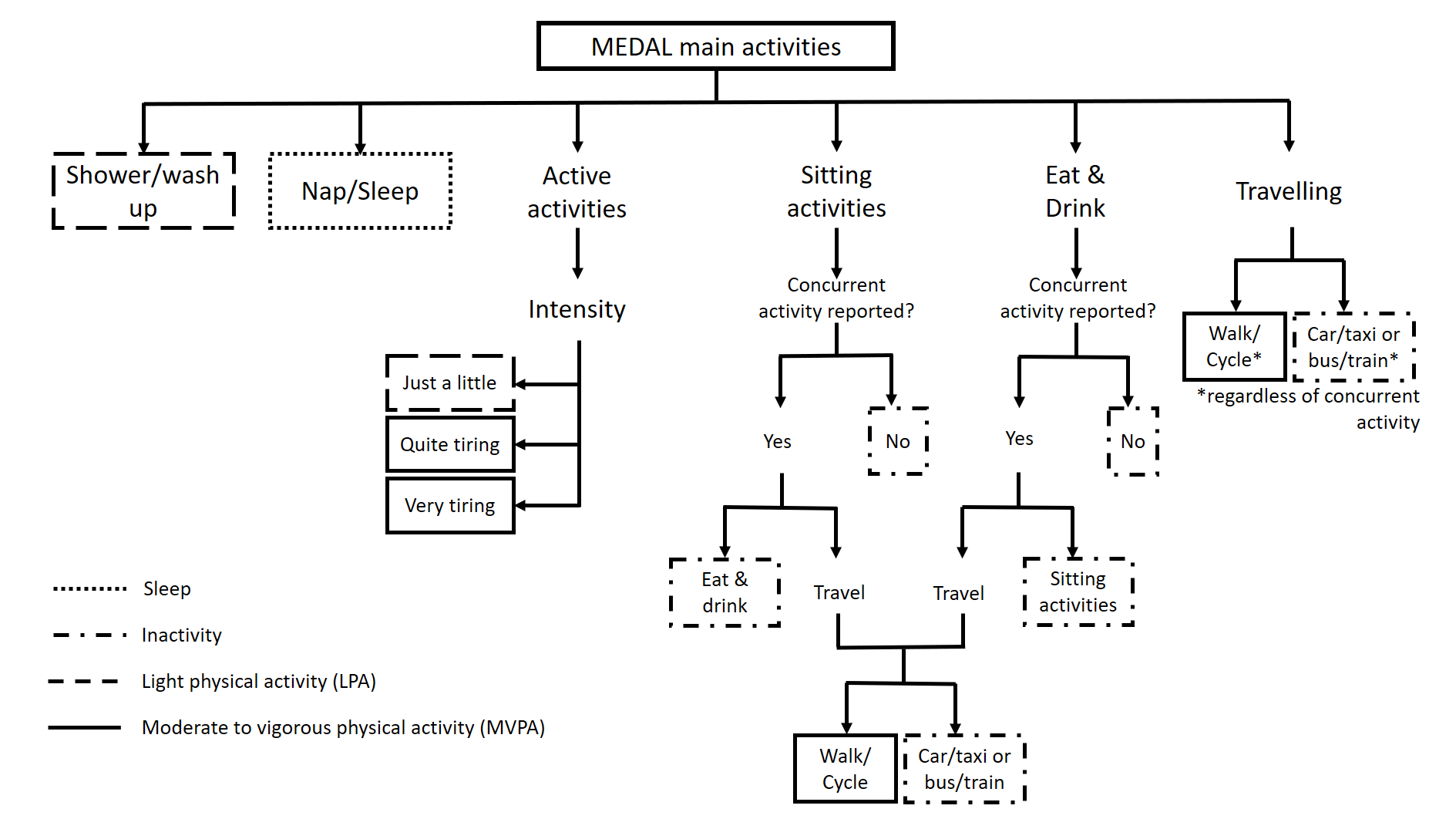


**Multimedia Appendix 1.** Classification of activities reported on MEDAL as inactivity, light physical activity, moderate-to-vigorous physical activity and sleep
