# Supplementary material for: A Web-Based, Time-Use App To Assess Children’s Movement Behaviors: Validation Study of My E-Diary for Activities and Lifestyle (MEDAL)
Source: JMIR Pediatr Parent. 2022 Jun 24;5(2):e33312. doi: 10.2196/33312 (PMC9270708; doi:10.2196/33312)
Supplement: Multimedia Appendix 2 [file pediatrics_v5i2e33312_app2.docx]

**Multimedia Appendix 2.** Demographic characteristics of Primary 5 students included and those recruited but were not included in the MEDAL movement behaviours validation study

|  | **Included (n=49)^a^** | **Not included (n=179)^b,c^** | ***P* value^d^** |
| --- | --- | --- | --- |
| Age, n (%) |  |  | .224 |
| *10 years* | 20 (40.8) | 94 (54.0) |  |
| *11 years* | 28 (57.1) | 78 (44.8) |  |
| *12 years* | 1 (2.0) | 1 (0.6) |  |
| *13 years* | 0 (0.0) | 1 (0.6) |  |
| School, n (%) |  |  | 0.077 |
| *School A* | 29 (59.2) | 131 (73.2) |  |
| *School B* | 20 (40.8) | 48 (26.8) |  |
| Sex, n (%) |  |  | 1.000 |
| *Females* | 20 (40.8) | 73 (40.8) |  |
| *Males* | 29 (59.2) | 106 (59.2) |  |
| BMI-for-age, n (%) |  |  | .149 |
| *Underweight* | 10 (20.8) | 18 (10.3) |  |
| *Healthy* | 33 (68.8) | 127 (73.0) |  |
| *Overweight* | 5 (10.4) | 29 (16.7) |  |
| Internet access, n (%) |  |  | .035* |
| *Yes* | 43 (97.7) | 142 (85.0) |  |
| *No* | 1 (2.3) | 25 (15.0) |  |

^a^Among included participants, one participant had missing BMI-for-age information, and five participants had missing information on internet access

^b^Participants who were not included refers to those who were either excluded from the movement behaviour validation analyses or those involved in the other validation studies

^c^Among the not included participants, five participants had missing information on age, five participants had missing BMI-for-age information, and 12 participants had missing information on internet access

^d^Difference in distribution of characteristics were assessed by Fisher’s exact test.

An asterisk (*) denoted statistical significance at 5% level of significance.
